# Viremia and HIV Drug Resistance Among People Receiving Dolutegravir Versus Efavirenz-Based First-Line Antiretroviral Therapy

**DOI:** 10.1097/QAI.0000000000003385

**Published:** 2024-03-11

**Authors:** Jienchi Dorward, Yukteshwar Sookrajh, Richard Lessells, Elliot Bulo, Nicola Bodley, Lavanya Singh, Pravikrishnen Moodley, Natasha Samsunder, Paul K. Drain, Gail Hayward, Christopher C. Butler, Nigel Garrett

**Affiliations:** a Nuffield Department of Primary Care Health Sciences, University of Oxford, Oxford, United Kingdom;; b Centre for the AIDS Programme of Research in South Africa (CAPRISA), University of KwaZulu–Natal, Durban, South Africa;; c eThekwini Municipality Health Unit, Durban, South Africa;; d KwaZulu-Natal Research and Innovation Sequencing Platform (KRISP), University of KwaZulu-Natal, Durban, South Africa;; e Department of Virology, University of KwaZulu-Natal and National Health Laboratory Service, Inkosi Albert Luthuli Central Hospital, KwaZulu-Natal, South Africa;; f Department of Global Health, Schools of Medicine and Public Health, University of Washington, Seattle, WA;; g Department of Medicine, School of Medicine, University of Washington, Seattle, WA;; h Department of Epidemiology, School of Public Health, University of Washington, Seattle, WA; and; i Discipline of Public Health Medicine, School of Nursing and Public Health, University of KwaZulu-Natal, Durban, South Africa.

## Abstract

Supplemental Digital Content is Available in the Text.

***To the Editors:***

## INTRODUCTION

Dolutegravir, an integrase strand transfer inhibitor, is currently being rolled out across low- and middle-income countries (LMICs).^1,2^ It has shown better effectiveness, tolerability, and has a higher genetic barrier to drug resistance compared with previous nonnucleoside reverse transcriptase (NNRTI)-based regimens such as efavirenz.^3^ People with viremia receiving dolutegravir may be more likely to have inconsistent adherence than HIV drug resistance (HIVDR). However, the absence of widespread HIVDR testing in LMICs^4^ makes it difficult for clinicians to determine the cause of viremia and manage it appropriately.

Among people receiving NNRTIs with viral failure (2 consecutive viral loads [VLs] ≥1000 copies/mL, ≥3 months apart), approximately 70% have drug resistance, and therefore, current World Health Organization guidelines recommend switching to second-line antiretroviral therapy (ART).^5,6^ Current World Health Organization guidelines for managing viremia on first-line dolutegravir are less clear because there is little data from LMICs regarding dolutegravir drug resistance and subsequent VL outcomes.

Therefore, among people with viremia on dolutegravir- and efavirenz-based first-line ART, we aimed to compare subsequent VL trajectories and drug resistance profiles.

## METHODS AND ANALYSIS

We used data from the POwER study, a randomized study of point-of-care VL testing among people with HIV viremia receiving first-line ART. The protocol and results have been previously published.^7,8^

### Setting and Participants

POwER was conducted at 2 public clinics in KwaZulu-Natal, South Africa, where dolutegravir has been recommended for first- and second-line ART from December 2019.^9^ Clinical management in POwER followed South African guidelines, which at the time recommended that people with viremia ≥1000 copies/mL should receive enhanced adherence counselling, with a repeat 3-month VL. If this remained high, those receiving efavirenz were recommended to switch to second-line ART, whereas those receiving dolutegravir were recommended to continue enhanced adherence counselling and repeat VL testing. Eligibility criteria for POwER were being ≥18 years old, nonpregnant, and receiving first-line dolutegravir or efavirenz-based ART, with viremia ≥1000 copies/mL in the past 6 weeks and yet to receive enhanced adherence counselling. Dolutegravir recipients may have been initiated on dolutegravir or previously transitioned from efavirenz.

### Procedures

Consenting participants were enrolled, received enhanced adherence counselling,^10^ and were randomized to point-of-care or standard laboratory-based VL testing after 12 weeks. Management of these VL results and clinical care during the 24 weeks of follow-up was provided by public sector healthcare workers. Plasma samples were stored at enrolment, 12-week VL, and 24-week study exit visits, for retrospective VL and drug resistance testing, with results not used for clinical management. All samples with VL ≥500 copies/mL were sequenced using next-generation sequencing with the Illumina MiSeq platform (Illumina, San Diego, CA) (see Supplemental Digital Content, http://links.lww.com/QAI/C216). We identified major drug resistance mutations (DRMs) at >20% frequency in protease, integrase, and reverse transcriptase regions using the Stanford HIVDR database.

### Variables and Analyses

The main exposure was dolutegravir- or efavirenz-based ART at enrolment. We conducted descriptive analyses and used Fisher exact test to assess the proportions in each ART group who had viremia ≥1000 copies/mL at enrolment, 12 weeks, and 24 weeks. We also assessed the proportions with HIVDR at each time point and switched to second-line ART.

### Ethical Approvals

The University of KwaZulu-Natal Biomedical Research Ethics Committee (BREC 00000836/2019) and the University of Oxford Tropical Research Ethics Committee (OxTREC 66-19) approved the study.

## RESULTS

### Participants

We enrolled 80 eligible participants between August 2020 and March 2022, an estimated 23.7% of those who were potentially eligible at the study clinics.^8^ Median age was 38.5 years (interquartile range [IQR] 33–45), 58.8% were female, and median time on ART was 3.2 years (IQR 1.0–6.0) (Table S1, Supplemental Digital Content, http://links.lww.com/QAI/C216).

At enrolment, 37 participants (46.3%) had been receiving efavirenz-based first-line regimens for a median of 3.2 years (1.1–5.0), and 43 (53.7%) had been receiving dolutegravir for a median of 0.7 years (IQR 0.5–1.1). Of the 43 participants, 15 (34.9%) had been initiated on dolutegravir, whereas 28 (65.1%) had been initiated on an efavirenz-based regimen and were subsequently transitioned to first-line dolutegravir. The dolutegravir group had less time on ART, slightly higher incomes and higher CD4 counts, but otherwise were similar to the efavirenz group (Table S1, Supplemental Digital Content, http://links.lww.com/QAI/C216). All participants were receiving tenofovir disoproxil fumarate, apart from 1 dolutegravir participant receiving abacavir.

### Viremia and HIV Drug Resistance

#### Enrolment

The median time since the pre-enrolment VL of ≥1000 copies/mL to enrolment was around 2 weeks (Table 1). At enrolment, the numbers with viremia ≥1000 copies/mL had fallen to 18 of 43 (41.9%) dolutegravir participants compared with 27 of 37 (73.0%) efavirenz participants (*P* = 0.007). HIVDR testing was attempted in all 45 participants with viremia ≥1000 copies/mL, and an additional 5 participants with VLs between 500 and 999 copies/mL. Of these 50 participants, HIVDR testing was successful in 48 for reverse transcriptase and 47 for integrase. The proportion with DRMs against either of the nucleoside reverse transcriptase inhibitor (NRTI) backbone drugs was lower in dolutegravir participants (2/19, 10.5%, 95% CI: 1.9 to 32.9) compared with efavirenz participants (21/29, 72.4%, 54.0, 85.4, *P* ≤ 0.001, Table 1). In efavirenz participants, 25 of 29 (86.2%, 68.7, 95.0) had DRMs against efavirenz, whereas among dolutegravir participants, there were no DRMs against dolutegravir.

**TABLE 1.** Outcomes Among Patients With Viremia on Dolutegravir- and Efavirenz-Based First-Line ART

Variable	Levels	Dolutegravir, n = 43	Efavirenz, n = 37^*^	*P*
Enrolment				
Days since pre-enrolment viral load ≥1000 copies/mL	Median (IQR)	16.0 (13.5–20.0)	14.0 (13.0–21.0)	0.333
Enrolment viral load, copies/mL	<1000 copies/mL	25 (58.1)	10 (27.0)	0.007
	≥1000 copies/mL	18 (41.9)	27 (73.0)	
Predicted active NRTIs in current regimen^†^	0	1 (5.3)	12 (41.4)	<0.001
	1	1 (5.3)	9 (31.0)	
	2	17 (89.5)	8 (27.6)	
Predicted active dolutegravir or efavirenz in the current regimen^†^	No	0 (0.0)	25 (86.2)	<0.001
	Yes	18 (100.0)	4 (13.8)	
Week 12 follow-up				
Time to follow-up viral load, days	Median (IQR)	91.0 (84.0–98.0)	90.5 (84.0–98.0)	0.613
Follow-up viral load, copies/mL^‡^	<1000 copies/mL	34 (85.0)	14 (37.8)	<0.001
	≥1000 copies/mL	6 (15.0)	23 (62.2)	
Predicted active NRTIs in current regimen^§^	0	0 (0.0)	13 (61.9)	<0.001
	1	0 (0.0)	6 (28.6)	
	2	6 (100.0)	2 (9.5)	
Predicted active dolutegravir or efavirenz in current regimen§^§^	No	0 (0.0)	21 (100.0)	
	Yes	6 (100.0)	0 (0.0)	
ART regimen change during follow-up?	No	43 (100.0)	6 (16.2)	<0.001
	Yes		31 (83.8)	
Reason for ART regimen change	ART policy change		7 (22.6)	1.000
	Virologic failure		24 (77.4)^‖^	
New ART regimen	AZT/3TC/DTG		17 (54.8)	1.000
	AZT/3TC/LPVr		2 (6.5)	
	TDF/3TC/DTG		7 (22.6)	
	TDF/AZT/3TC/DTG^¶^		4 (12.9)	
	TDF/FTC/LPVr		1 (3.2)	
Week 24 exit				
Exit viral load, copies/mL^#^	<1000 copies/mL	34 (85.0)	33 (94.3)	0.271
	≥1000 copies/mL	6 (15.0)	2 (5.7)	
Predicted active NRTIs in current regimen^**^	0	1 (20.0)	2 (66.7)	
	1	0 (0.0)	1 (33.3)	
	2	4 (80.0)	0 (0.0)	
Predicted active dolutegravir or efavirenz in current regimen^**^	No	0 (0.0)	0 (0.0)	
	Yes	7 (100.0)	3 (100.0)	

* One participant had been transitioned from TDF/FTC/EFV to TDF/3TC/DTG 15 days before enrolment, on the same day of the pre-enrolment viral load. At the enrolment visit, they were changed back to TDF/FTC/EFV because they should not have been transitioned while viremic, with VL >1000 copies/mL. Their 12-week follow-up viral load was 1222 copies/mL, and so they were switched from TDF/FTC/EFV to second-line AZT/3TC/LPVr.

† 50 participants had viral load >500 copies/mL and HIVDR testing was successful in 48 for reverse transcriptase and 47 for integrase.

‡ 3 participants in the dolutegravir group had no follow-up viral load.

§ 32 participants had viral load >500 copies/mL and HIVDR testing was successful in 27 for reverse transcriptase and 30 for integrase.

‖ 1 participant with repeat viral load of 937 copies/mL was deemed by the clinician to have virologic failure and switched to second-line AZT/3TC/DTG.

¶ Remained on tenofovir due to Hepatitis B infection.

# 2 participants in each group were lost to follow-up, and 1 dolutegravir participant had no exit viral load.

** 10 participants had viral load >500 copies/mL and HIVDR testing was successful in 8 for reverse transcriptase and 10 for integrase.

AZT, zidovudine; DTG, dolutegravir; EFV, efavirenz; FTC, emtricitabine; TDF, tenofovir disoproxil fumarate; LPVr, lopinavir/ritonavir.

#### Follow-up

By the time of the 12-week VL, participants in both the dolutegravir and the efavirenz groups had a median of 1 (IQR 1, 1) enhanced adherence counselling sessions. Only 6 of 43 (15.0%) of dolutegravir participants had a VL ≥1000 copies/mL and were classified as having viral failure compared with 23 of 37 (62.2%) efavirenz participants (*P* < 0.001). All 23 efavirenz participants with confirmed viral failure at 12 weeks, and 1 other with a repeat VL of 937 copies/mL, were switched to second-line regimens (Tables S1 and S2, Supplemental Digital Content, http://links.lww.com/QAI/C216), at a median of 90 days (IQR 84–99) after enrolment. The commonest second-line regimen was zidovudine, lamivudine, and dolutegravir. Overall, 32 participants had 12-week VLs >500 copies/mL, and of these, HIVDR testing was successful in 27 participants for reverse transcriptase and 30 participants for integrase. None of the 6 dolutegravir participants had dolutegravir or NRTI DRMs compared with 19 of 21 (90.5%, 69.6, 98.4) efavirenz participants who had resistance against the NRTI backbone (*P* < 0.001); 21 of 21 (100%, 81.4, 100) had resistance against efavirenz.

At the 24-week exit visit, 2 participants in each group were lost to follow-up, and 1 dolutegravir participant had no exit viral load taken. Of those with exit viral loads, viremia was detected in 6 of 40 participants (15.0%) who were receiving dolutegravir at enrolment, versus 2 of 35 (5.7%) of those who were receiving efavirenz at enrolment (*P* = 0.271). Among the 8 of 10 with successful NRTI HIVDR testing, 1 of 5 (20.0%, 2.5, 64.1) in the dolutegravir enrolment group had resistance against the NRTI backbone versus 3 of 3 (100%, 40.0, 100) in the efavirenz enrolment group. One participant who was receiving tenofovir disoproxil fumarate, lamivudine and dolutegravir from enrolment, and had only NNRTI DRMs at enrolment, developed an emergent K65R mutation by week 24 (Table S2, Supplemental Digital Content, http://links.lww.com/QAI/C216). There were no dolutegravir DRMs detected from enrolment to study exit in any participants.

## DISCUSSION

At enrolment and 12-week follow-up, people receiving efavirenz-based ART with viremia had high levels of DRMs against their first-line regimen, whereas people receiving dolutegravir had minimal resistance. Consequently, dolutegravir participants had higher levels of resuppression at 12 weeks compared with efavirenz. After switching to second-line ART, 24-week viral resuppression in efavirenz participants became similar to dolutegravir, with few DRMs in both groups.

Among participants receiving dolutegravir-based ART at baseline, there were no integrase strand transfer inhibitor mutations, meaning that viremia was likely caused by poor adherence. Our study is one of the first to report outcomes among people experiencing viremia on first-line dolutegravir in LMICs, with 85.0% achieving viral suppression <1000 copies/mL after 12 weeks. In contrast, a high proportion of participants receiving efavirenz-based ART had baseline NRTI and NNRTI resistance, meaning resistance was contributing to viremia. After 12 weeks, 37.8% resuppressed to <1000 copies/mL, similar to the 46.4% among people receiving NNRTI-based ART in a large systematic review.^11^ The remaining participants only resuppressed after switching to second-line ART. One other study compares resuppression among people with viremia receiving dolutegravir versus efavirenz in LMICs.^12^ Among people with viremia after initiating ART in the ADVANCE trial, resuppression was more frequent in the dolutegravir group (155/247, 62.8%) compared with efavirenz (44/138, 32%, *P* <0.001).^12^ There was 1 case of emergent resistance to dolutegravir.

Strengths of our study include the focus on people with viremia while receiving dolutegravir, successful HIVDR testing in a high proportion of those with viremia, and frequent VL testing. The small sample size meant we could not adjust for potential confounding factors that could contribute to the difference in outcomes between dolutegravir and efavirenz participants. For example, people who were transitioned to dolutegravir may be better engaged in care or motivated to adhere to treatment, and therefore also more likely to resuppress. Follow-up time was short, and the median time on dolutegravir was less than a year.

Nevertheless, our findings, alongside those of the ADVANCE study, demonstrate that early in the South African rollout, viremia among people receiving dolutegravir is largely because of poor adherence rather than drug resistance. This supports the current South African guidelines, which do not recommend early switching to second-line ART or routine HIVDR testing among people receiving first-line dolutegravir with viral failure. The World Health Organization does not currently have specific guidance for management of viral failure in people receiving first-line dolutegravir. Further evidence is needed to determine the extent and impact of emergent DRMs with long-term viremia on dolutegravir. In the meantime, managing viremia among people receiving dolutegravir should have a renewed focus on interventions to support adherence rather than managing HIVDR.

## Supplementary Material
